# Prevalence of chronic hepatitis B virus infection and infrastructure for its diagnosis in Madagascar: implication for the WHO’s elimination strategy

**DOI:** 10.1186/s12889-017-4630-z

**Published:** 2017-08-04

**Authors:** Soa Fy Andriamandimby, Marie-Marie Olive, Yusuke Shimakawa, Fanjasoa Rakotomanana, Iony Manitra Razanajatovo, Tsarasoa Malala Andrianinarivomanana, Jean-Pierre Ravalohery, Seta Andriamamonjy, Christophe Rogier, Jean-Michel Héraud

**Affiliations:** 1 Virology Unit, Institut Pasteur of Madagascar, Antananarivo, Madagascar; 2 Epidemiology Unit, Institut Pasteur of Madagascar, Antananarivo, Madagascar; 3 Direction centrale du service de santé des armées Parcelle, Paris, France; 4 Unité d’Epidémiologie des Maladie Emergentes, Institut Pasteur, Paris, France; 5 UPR AGIRs, CIRAD Montpellier, Montpellier, France

## Abstract

**Background:**

WHO developed a global strategy to eliminate hepatitis B by 2030 and set target to treat 80% of people with chronic hepatitis B virus (HBV) infection eligible for antiviral treatment. As a first step to achieve this goal, it is essential to conduct a situation analysis that is fundamental to designing national hepatitis plans. We therefore estimated the prevalence of chronic HBV infection, and described the existing infrastructure for HBV diagnosis in Madagascar.

**Methods:**

We conducted a stratified multi-stage serosurvey of hepatitis B surface antigen (HBsAg) in adults aged ≥18 years using 28 sentinel surveillance sites located throughout the country. We obtained the list of facilities performing HBV testing from the Ministry of Health, and contacted the person responsible at each facility.

**Results:**

A total of 1778 adults were recruited from the 28 study areas. The overall weighted seroprevalence of HBsAg was 6.9% (95% CI: 5.6–8.6). Populations with a low socio-economic status and those living in rural areas had a significantly higher seroprevalence of HBsAg. The ratio of facilities equipped to perform HBsAg tests per 100,000 inhabitants was 1.02 in the capital city of Antananarivo and 0.21 outside the capital. There were no facilities with the capacity to perform HBV DNA testing or transient elastography to measure liver fibrosis. There are only five hepatologists in Madagascar.

**Conclusion:**

Madagascar has a high-intermediate level of endemicity for HBV infection with a severely limited capacity for its diagnosis and treatment. Higher HBsAg prevalence in rural or underprivileged populations underlines the importance of a public health approach to decentralize the management of chronic HBV carriers in Madagascar by using simple and low-cost diagnostic tools.

**Electronic supplementary material:**

The online version of this article (doi:10.1186/s12889-017-4630-z) contains supplementary material, which is available to authorized users.

## Background

Hepatitis B virus (HBV) is a DNA virus belonging to the *Hepadnaviridae* family. It is estimated that at least two billion people have been infected with HBV, and 240 million individuals suffer from chronic HBV infections [1]. Chronic HBV infection is a major risk factor for liver disease, including liver cancer [2], one of the most frequent malignancies in Africa [3]. Hepatitis B has been largely ignored as an important global health priority until recently [4], despite the heavy disease burden. In 2015, the United Nations adopted the resolution in the 2030 Agenda for Sustainable Development, in which viral hepatitis became one of the targeted infectious diseases within the United Nations’ development goals. Subsequently, the World Health Organization (WHO) has developed a strategy to eliminate HBV as a public health threat by 2030, aiming to reduce the incidence of new chronic infections by 90% and HBV-related mortality by 65%. Increasing the coverage of HBV testing and antiviral treatment became one of the important targets [4], and the feasibility of a community-based HBV screening and linkage to care with antiviral therapy in resource-limited settings was recently demonstrated by the PROLIFICA project in West Africa [5].

Madagascar is a large island located in the Indian Ocean close to the southeast of the African continent, with 23.5 million inhabitants. Although the hepatitis B vaccine was integrated into the national immunization program in 2002, vaccination is only provided as part of the pentavalent vaccine given after the age of 6 weeks, without scheduling a dose at birth. Moreover, the coverage of three doses of hepatitis B vaccine in infants was estimated at 69% in 2015, which is inadequate [6]. There is no national hepatitis plan or program to screen, diagnose, or treat chronic HBV carriers. A recent systematic review of the global seroprevalence of HBsAg using data from seven previous serosurveys reported a pooled estimate of 4.6% (95% CI: 4.4–4.8%) in Madagascar [1]. However, these surveys were limited because they only sampled participants from one or two locations in the country [7–14].

As a first step to achieve the WHO’s global elimination strategy, it is essential to better estimate HBV prevalence in the population before designing national strategies. We therefore conducted a country-wide serosurvey to estimate the seroprevalence of HBsAg in adults in Madagascar and to identify factors associated with it. We also described the current infrastructure for HBV testing and treatment in the country.

## Methods

### Study population

**Fig. 1:**
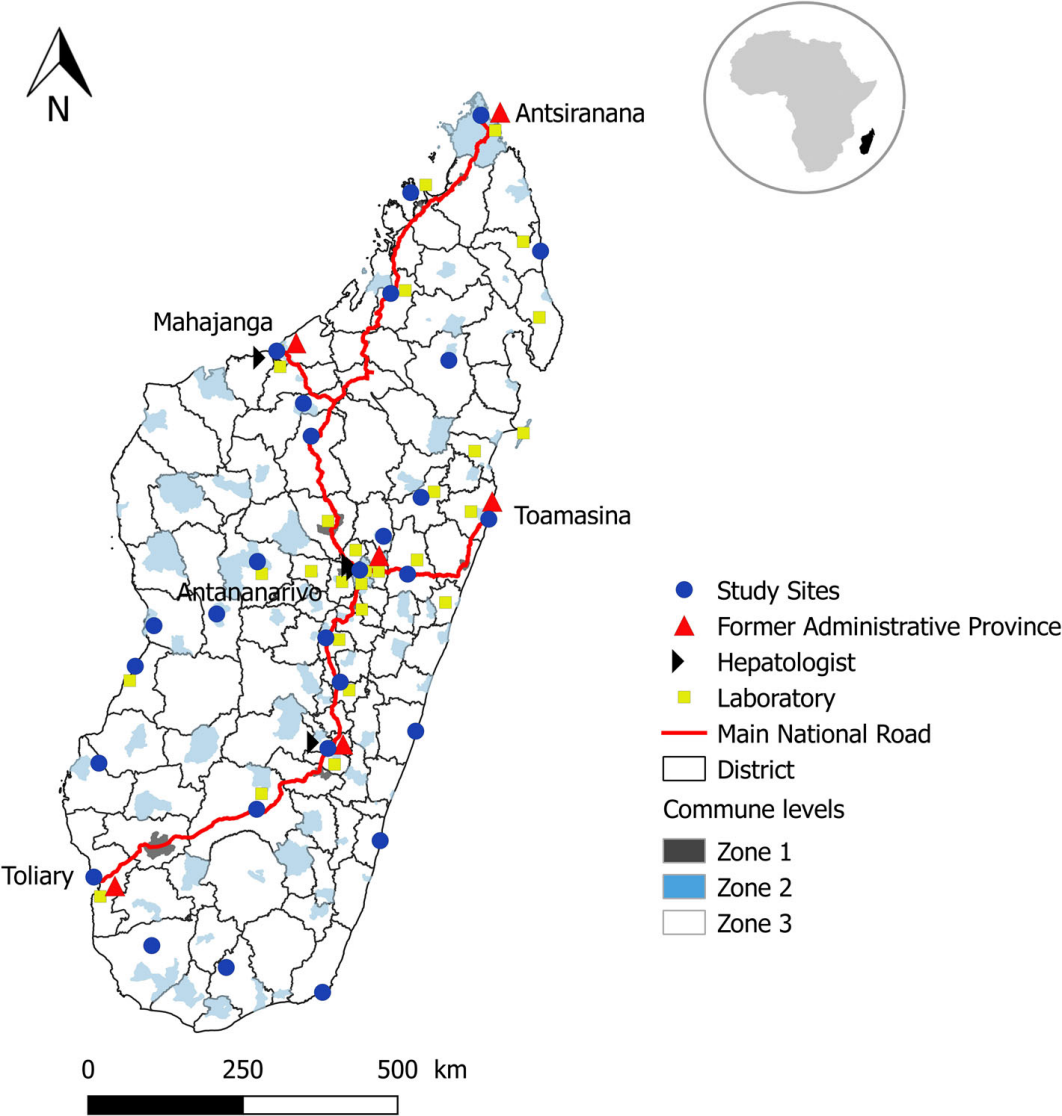
Map of study sites in Madagascar describing the different areas defined as Zone 1 for urban (in *grey*), Zone 2 for sub-urban (in *blue*) and Zone 3 for rural (in *white*)

We conducted a stratified multi-stage sample serosurvey between November 2011 and May 2013. We used sentinel surveillance sites for febrile illness as a primary sampling unit to obtain representative samples of the general population in Madagascar, because these are evenly scattered throughout the country [15]. We excluded three of 31 health centres that participate in this surveillance system (Maroantsetra, Sainte Marie, and Maintirano) because access to these sites required air travel. The remaining 28 sites were used to select study villages (Fig. 1). We stratified the coverage area of each of the 28 health centres into ‘central’ and ‘peripheral’ depending on the distance from the health centre. Then, by using a random number generator (Excel, Microsoft) we randomly selected one village from a central area and another from a peripheral area. Overall, we assessed 28 central villages and 28 peripheral villages. We randomly selected households in each village, and the team of fieldworkers visited these selected households until they could recruit at least 31 adults aged ≥18 years.

Following written informed consent, we administered a standardised epidemiological questionnaire and obtained a venous blood sample from study participants. Collected serum samples were transported to the Virology Unit of the Pasteur Institute of Madagascar, and stored at −80 °C.

### Serological assay

We detected HBsAg using an in-house ELISA that was validated at our Virology unit. The in-house assay had good diagnostic accuracy for the detection of HBsAg (sensitivity, 95.6% (95% CI, [78.05, 100]; specificity, 100% (95% CI, [80.36, 100], using a commercial chemiluminescent microparticle immunoassay (Architect®, Abbott, USA) as a reference. Briefly, for the in-house capture-ELISA, a goat polyclonal anti-HBs antibody (Reference ABIN731303, Germany) was first used. After binding of the sera, a second goat polyclonal (biotin) antibody (Reference ABIN731305, Germany) was used. At the end of the reaction, Streptavidin-HRP conjugate (Reference RPN 1231 - AEC Amersham) was added before the revelation step using ABTS® (Sigma-Aldrich).

### Sample size calculation

Assuming that the expected seroprevalence of HBsAg is 10% and the desired width of the 95% confidence interval is ±2% with a design effect of 2.0 [16], the minimum sample size required was 1729. We recruited at least 31 adults per village since there were 56 villages studied.

### Data analysis

Statistical analyses were performed using STATA IC. 11.0 (Stata Corporation, USA). The seroprevalence of HBsAg was estimated by dividing the number of HBsAg-positive people by the number of survey participants. The estimates were weighted for sampling probability and adjusted for stratification by central or peripheral area and clustering in the survey design, using the “svy” command in STATA. Similarly, a weighted logistic regression, that accounted for stratification and clustering by village, was used to identify factors associated with HBsAg seropositivity. The factors of interest included basic demographic variables (age and sex) and potential barriers to linkage from HBsAg screening to care (education levels, socio-economic status (SES), and area of residency) [17–19]. After univariable analysis, the associations between these covariates and HBsAg seropositivity were adjusted for age and sex in multivariable logistic regression models.

We used principal component analysis (PCA) and hierarchical cluster analysis (HCA) to define the SES of each individual. We included the following variables known to represent the SES in the PCA: type of wall, floor, and roof material; type of energy for light and cooking; sanitation facility; and main assets [20–22]. Hierarchical cluster analysis was performed using raw individual weights obtained from preliminary PCA using Ward method. Consolidation was performed using k-means methods. We classified the villages into three groups: urban, semi-urban, and rural (Fig. 1). The urban area was defined to be within an administrative centre of a district and located on the main road that connects the main Provinces to the capital Antananarivo. The semi-urban area is also within an administrative centre of a district, but not on the main national road. We categorised a village as rural when none of the above criteria were met. There is at least one regional hospital in the urban areas, whereas there is at least one district hospital in the semi-urban areas. There are no hospitals in the rural areas; only a primary health care centre.

### Infrastructure for HBV testing/treatment in Madagascar

We determined the number of laboratories or health facilities that routinely carry out each of the following tests essential for HBV screening and clinical staging: HBsAg serology, determination of alanine (ALT) and aspartate transaminase (AST) levels, detection or quantification of HBV DNA, transient elastography (Fibroscan®), and liver biopsy. We also determined the number of hepatologists and pharmacies that routinely dispense antiviral drugs (lamivudine or tenofovir). In April 2016, we first requested the Ministry of Health to generate a list of facilities including information on each of these items. Subsequently we verified the list by contacting a person responsible at each facility using a standardised questionnaire. We also gathered data on the cost of each service. We presented the ratio of the number of facilities with the capacity to test individuals for chronic HBV infection per 100,000 inhabitants, a method proposed by the WHO [23].

## Results

### Seroprevalence of HBsAg

A total of 1778 participants were recruited from the 28 study areas. Approximately half (50.6%) were men. The mean age was 37.7 years (SD ± 14.4) and ranged from 18 to 99 years. The overall weighted seroprevalence of HBsAg was 6.9%, 95% CI [5.6–8.6] (141/1778). The prevalence varied markedly between villages, ranging from 0.0% to 26.7%.

### Factors associated with HBsAg seropositivity

**Fig. 2:**
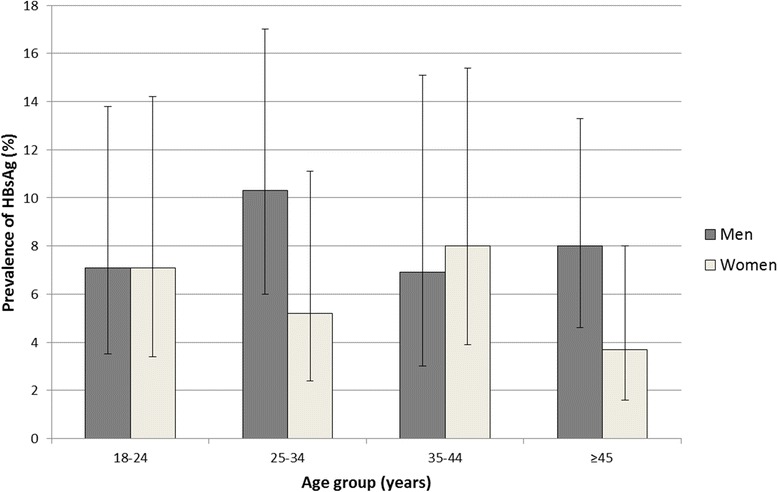
The age- and sex-specific prevalence of HBsAg seropositivity in Madagascar, 2011–2013

Table 1 presents the factors associated with HBsAg seropositivity. There was no clear association between either age group or sex and the seroprevalence of HBsAg. The seroprevalence of HBsAg in women of childbearing age (18–45 years old) was 6.2%, 95% CI [3.4–8.9] (46/630) (Fig. 2).

**Table 1:** Factors associated with HBsAg seropositivity in Madagascar, 2011–2013

	N	HBsAg- positive, n (weighted %)	Crude odds ratios		Odds ratios adjusted for age and sex	
			OR (95% CI)	*p*-value	OR (95% CI)	*p*-value
Age (years)				0.5*		0.4*
18–24	380	38 (7.1)	1.00		1.00	
25–34	475	41 (7.4)	1.05 (0.50–2.22)		1.08 (0.50–2.34)	
35–44	379	26 (7.5)	1.06 (0.58–1.97)		1.09 (0.59–2.01)	
≥ 45	542	36 (6.0)	0.84 (0.42–1.65)		0.82 (0.42–1.61)	
Gender				0.3		0.3
Female	879	58 (5.8)	1.00		1.00	
Male	899	83 (8.1)	1.42 (0.75–2.68)		1.43 (0.76–2.67)	
Education				0.7*		0.4*
Illiterate	233	18 (5.5)	1.00		1.00	
Primary	668	62 (8.6)	1.62 (0.81–3.21)		1.62 (0.80–3.25)	
Secondary	794	49 (5.2)	0.96 (0.47–1.96)		0.91 (0.44–1.88)	
University	81	12 (13.5)	2.70 (1.05–6.97)		2.48 (0.93–6.61)	
SES				0.03*		0.03*
Low	935	81 (9.4)	1.00		1.00	
Middle	753	57 (6.6)	0.69 (0.41–1.15)		0.70 (0.42–1.17)	
High	90	3 (3.8)	0.38 (0.13–1.10)		0.39 (0.14–1.11)	
Area				0.04*		0.04*
Urban	482	26 (6.0)	1.00		1.00	
Semi-urban	516	39 (7.2)	1.22 (0.70–2.12)		1.23 (0.71–2.11)	
Rural	780	76 (9.8)	1.71 (1.11–2.64)		1.69 (1.09–2.60)	

*The *p*-value was derived from a test for trends

Variable characterizing low socio economical level were mainly wooden combustion use, roof made in plants, light of the petroleum lamp, dirt floor in the bedroom and not equipped with toilet (v-test were respectively 31.0, 30.5, 26.05, 24.02 and 22.16); variables characterizing intermediated socio-economical level were wood charcoal combustion, sheet roof, electricity light, TV and cement floor (v-test = 26.2, 25.9, 23.5, 22.9 and 22.0, respectively); variables characterizing the highest socio-economical level were computer owning, flash toilet owning, internet access, car and refrigerator owning (v-test were respectively 31.9, 26.5, 26.0, 22.4 and, 21.8). Repartition of characteristic variables in each SES level were described in Additional file 1: Table S1. Of the three potential barriers to linkage to care, low SES and living in a rural area were significantly associated with HBsAg seropositivity after adjusting for age and sex. The middle and high SES groups had a seroprevelance of HBsAg that was 0.70 [0.42–1.17] and 0.39 [0.14–1.11] times, respectively, that of the low category SES group (adjusted p for Wald test for trend = 0.03). Using the participants living in urban areas as the reference, the odds ratios were 1.23 [0.71–2.11] for semi-urban and 1.69 [1.09–2.60] for rural areas (adjusted *p* for Wald test for trend = 0.04).

### Infrastructure for HBV testing/treatment

Table 2 summarises the current infrastructure for HBV screening, clinical staging, and treatment in Madagascar. There were 77 laboratories routinely equipped to perform HBsAg serology and liver enzyme tests (AST and ALT). Although many have the capacity to perform ELISA tests, only three laboratories in the capital routinely performed ELISA assays for HBsAg detection, whereas the rest used immunochromatographic point-of-care assays. These 77 facilities cover only 31 of the 114 administrative districts. Thirty-six (46.8%) of the laboratories are located in the capital, resulting in a ratio of 1.03 facilities per 100,000 inhabitants in the capital region and 0.21 per 100,000 inhabitants outside. Furthermore, many of these laboratories are not fully functional throughout the year because of limited resources, frequent reagent shortages, and laboratory equipment failure.

**Table 2:** Infrastructure for HBV testing/treatment in Madagascar in 2016

		Total number	No. of facilities in the capital region (Antananarivo)		No. of facilities in other regions		Price
			Public	Private	Public	Private	
1. Screening for HBV							
HBsAg assay	ELISA	3	2	1	0	0	$ 10
	Point-of-care test	74	11	22	29	12	$ 2–5
2. Clinical staging							
AST & ALT		77	13	23	29	12	$ 2–5
HBV DNA PCR assay		0	0	0	0	0	N/A
Fibroscan®		0	0	0	0	0	N/A
Hepatologist		5	3	0	2	0	N/A
3. Treatment							
HBV treatment	Lamivudine	1	0	1	0	0	$ 0.7/dose
	Tenofovir	0	0	0	0	0	N/A

*Abbreviations*: *ELISA* enzyme-linked immunosorbent assay, *N/A* not applicable

There are only five hepatologists in the country (0.08 and 0.01 per 100,000 inhabitants in Antananarivo and outside of the capital region, respectively). Three are in the capital: two at the largest public medical centre and one at a peripheral public hospital. Two others are in two hospitals outside the capital region in Fianarantsoa and in Mahajanga. None of the hepatologists routinely perform liver biopsies, and only a few surgeons occasionally perform the procedure. There is no Fibroscan® device and none of laboratories performs PCR assays for HBV DNA. When HBV DNA measurement is requested, sera are shipped to a commercial laboratory in France from the Pasteur Institute of Madagascar. Lamivudine is available in only one pharmacy in the capital, and no pharmacy dispenses tenofovir, even though the national authority has approved it.

## Discussion

We conducted a stratified multi-stage sample serosurvey to estimate the seroprevalence of HBsAg in Madagascar. We found that: (i) Madagascar has a high-intermediate level of endemicity of HBV infection, according to the WHO classification [1], with a weighted prevalence of 6.9%; and (ii) people with a low SES and those living in rural areas are more likely to be HBsAg seropositive. We also revealed a severely limited capacity for the diagnosis and treatment of chronic HBV in Madagascar.

This is the first study that attempted to estimate the seroprevalence of HBsAg in Madagascar using a representative sample of the country. The prevalence was weighted to reflect the probability of being included in the survey [16]. There have been 12 serosurveys for HBsAg in Madagascar to date (Table 3). However, these only focused on high-risk groups or a few communities within the country. By pooling data from these studies, a country-specific prevalence was recently estimated by Schweitzer et al. (4.6%, 95% CI [4.4–4.8]), which was much lower than our estimate (6.9%, 95% CI [5.6–8.6]). Their pooled estimate was low because of a large influence from one large study of blood donors in the capital, Antananarivo, (*n* = 47,597) which reported a prevalence of 3.8% [13]. The prevalence in the capital is lower than in other regions [5], and the risk of HBV infection in blood donors is likely to differ from that of the general population, possibly leading to an erroneous estimate.

**Table 3:** Prevalence of HBsAg seropositivity in previous studies (*n* = 12) and the current study in Madagascar

Author	Year published	HBsAg assay	Province	Study population	Male (%)	Age	No. tested	No. positive	Prevalence of HBsAg
Oddou [11]	1972	Immunodifusion	Antananarivo	Blood donors	N/R	N/R	578	17	2.9%
Capdevieille [30]	1979	PHA	Antananarivo	Blood donors	N/R	N/R	4000	217	5.4%
				Patients with liver disease	N/R	N/R	100	43	43.0%
Ravaoarinoro [14]	1985	ELISA	Antananarivo	Blood donors	87.2%	19–63	172	4	2.3%
				Patients	55.4%	1–65	186	8	4.3%
Mathiot [31]	1987	Electrosyneresis	Antananarivo	High-risk population^a^	50.0%	N/R	178	7	3.9%
Genin [32]	1988	ELISA	Toamasina	High-risk population^a^	N/R	Mean 27 (14–66)	367	65	17.7%
			Antsiranana	High-risk population^a^			278	26	9.4%
			Toliara	High-risk population^a^			258	46	17.8%
			Mahajanga	High-risk population^a^			228	41	18.0%
Rasamindrakotroka [33]	1993	ELISA	Antananarivo	Blood donors	83.9%	Range: 18–60	1629	77	4.7%
Morvan [10]	1994	ELISA	Antananarivo	General population	52.9%	Mean 22.0 (1–86)	456	86	18.9%
			Toliara	General population	48.2%	Mean 21.1 (1–62)	197	60	30.5%
Boisier [7]	1996	ELISA	Antananarivo	General population	45.4%	Range: 1–94	243	13	5.3%
			Toamasina	General population			678	176	26.0%
Migliani [9]	2000	ELISA	Mahajanga	General population	41.9%	Median 22.5 (2–93)	654	93	14.2%
Dupinay [8]	2010	ELISA	Antsiranana	General population	53.5%	Median 29 (15–55)	563	48	8.5%
Randriamanantany [13]	2011	IC	Antananarivo	Blood donors	80.5%	Mean 33.3 ± 10.4	47,597	1810	3.8%
Randriamahazo [12]	2015	IC	Antananarivo	Pregnant women	0%	Mean 26.5 (14–44)	1050	20	1.9%
Andriamandimby	2016	ELISA	All provinces	General population	50.3%	Mean 37.7(± 14.4)	1778	141	6.9%^b^

^a^High-risk population includes prostitutes, prisoners and men who have sex with men

^b^Weighted

*Abbreviations*: *PHA* passive hemagglutination, *ELISA* enzyme-linked immunosorbent assay, *IC* immunochromatography, *N/R* not reported

To reduce HBV-related mortality in Madagascar, hepatitis B vaccination alone is insufficient because 6.9% of adults who established chronic infection before the introduction of hepatitis B vaccines will continue to carry elevated risk of dying from HBV-related liver diseases. Alternatively, population-wide screening and treatment of HBV may reduce these deaths, as suggested by a recent modelling study [24]. We assessed factors associated with HBsAg seropositivity to identify sub-groups of the population with an increased risk of chronic HBV infection. Such information may help in the planning of a future “HBV screen and treat” program in Madagascar. We found that the prevalence did not vary significantly according to age or sex. However, we identified that people with a low SES and those living in rural communities have high HBV prevalence (9.4% and 9.8%, respectively). This is problematic as both low SES and rural residence are known barriers to access to healthcare in sub-Saharan Africa [18, 19]. The WHO recommends a public health approach to scaling up HBV testing and treatment, by ensuring the “widest possible access to high-quality services at the population level” [25]. Our results support the importance of this approach, and suggest that special care must be taken to not overlook underprivileged people or those in rural areas, not only to be equitable, but also because of the higher HBV prevalence in these populations.

Although the current infrastructure for “HBV screen and treat” in Madagascar is very limited, we still believe that screening, clinical staging, and treatment of people with chronic HBV infection will become feasible through the use of alternative diagnostic tools, as recommended in the WHO treatment guidelines [25]. First, HBsAg detection by ELISA is only performed in three laboratories in the capital and none in other regions. However, immunochromatographic point-of-care tests are available in many laboratories in the capital region and to a lesser extent in rural areas at an affordable price (2–5 USD/test). The high diagnostic accuracy of HBsAg point-of-care tests has been confirmed under field conditions in sub-Saharan Africa [26]. Second, although there is no Fibroscan® and liver biopsy is rarely performed in Madagascar, many laboratories still have the capacity to test AST and ALT. The WHO recommends the use of simple non-invasive markers for fibrosis staging such as APRI or Fib-4, and these only require basic laboratory markers including transaminase (AST, ALT) and platelet count. Third, the lack of capacity to evaluate HBV DNA levels seems to be a problem as its measurement is essential to define treatment eligibility according to European or American guidelines [27, 28]. However, the recent WHO guidelines acknowledged the lack of access to HBV DNA testing in low- and middle-income countries, and defined separate treatment criteria for places where HBV DNA testing is not available. These alternative criteria do not depend on HBV viral load; they only require ALT levels and an APRI score [25]. Although tenofovir is currently unavailable in Madagascar, the cost of antivirals does not need to be an obstacle as its generic version (50–350 USD/year) can be used. The most serious obstacle may be the lack of a sufficient healthcare workforce; there are only 0.02 hepatologists/100,000 inhabitants. Task shifting of HBV clinical management from hepatologists to mid-level health workers (e.g., registered nurses) needs to be carefully considered.

Our study has limitations. First, although we attempted to provide an estimate that represents the entire country, we excluded three of 31 sentinel sites because of difficulties of access. However, the effect should be minimal as the number of inhabitants covered by these sites is relatively small. Second, we only studied people aged 18 years or above, and did not assess the prevalence in children. This was the requirement from the funder of this study and sampling of children was therefore, not authorized by the local IRB. It is to be noted that we are currently conducting a study focusing on prevention of HBV in children called NéoVac (Neonatal Vaccination against Hepatitis B in Africa), which attempts to understand potential barriers to implement timely birth dose vaccination in Madagascar, Senegal and Burkina Faso. Third, we did not provide care for those who were identified to be positive for HBsAg during the serosurvey. When the study was planned in 2011, this was not required by the national ethics committee.

## Conclusion

The WHO has set a target to treat 80% of eligible people with chronic HBV infection to eliminate hepatitis B as a public health threat by 2030; globally, only 8% of this population was estimated to be under treatment in 2015 [29]. It is crucial to adopt a public health approach that ensures the widest possible access to HBV screening and care at the population level to achieve this goal in resource-limited countries [4]. Our study demonstrated a higher seroprevalence of HBsAg in rural areas where the access to HBV testing is limited, and in people with a low SES who may be unable to pay for HBV testing and treatment. This clearly requires the need to decentralize the management of chronic HBV carriers in Madagascar by using simple and low-cost diagnostic tools. A similar exercise deserves to be carried out in other low- and middle-income countries as this may help in designing an evidence-based national hepatitis plan.

## Additional files

Additional file 1: Table S1. Characteristics of household according to socio-economical level defined by PCA followed by HCA. (DOCX 16 kb)

Additional file 2: This file includes supplementary data. (CSV 90 kb)

## Abbreviations

ALT: Alanine transferase
AST: Aspartate transferase
CDC: Center for disease control
CI: Confidence interval
DNA: Desoxyribonucleic acid
ELISA: enzyme-linked immunosorbent assay
HBsAg: Hepatitis B surface antigen
HBV: Hepatitis B Virus
HCA: hierarchical cluster analysis
IC: immunochromatography
PCA: principal component analysis
PHA: Passive hemmaglutination
PROLIFICA: Prevention of liver fibrosis and cancer in Africa
SES: socio-economic status
WHO: World health organization
ZORA: Zoonoses, rodent and arboviruses
