# Supplementary material for: Prevalence of chronic hepatitis B virus infection and infrastructure for its diagnosis in Madagascar: implication for the WHO’s elimination strategy
Source: BMC Public Health. 2017 Aug 4;17:636. doi: 10.1186/s12889-017-4630-z (PMC5544978; doi:10.1186/s12889-017-4630-z)
Supplement: Supplementary file 1 — Characteristics of household according to socio-economical level defined by PCA followed by HCA. (DOCX 16 kb) [file 12889_2017_4630_MOESM1_ESM.docx]

Additional file 1: **Table S1**: Characteristics of household according to socio-economical level defined by PCA followed by HCA

|  | Variables | Level 1 | Level 2 | Level 3 |
| --- | --- | --- | --- | --- |
| Characterisctic of wall | Wattle and daub | 80.2 | 19.6 | 0.2 |
|  | Vegetable | 73.2 | 26.8 | 0.0 |
|  | Brick | 37.8 | 54.4 | 7.8 |
|  | Concrete | 2.7 | 57.3 | 40.0 |
|  | Wood | 34.5 | 61.0 | 4.5 |
|  | Breeze block | 0.0 | 83.8 | 16.2 |
|  | Sheet metal | 15.7 | 83.3 | 1.0 |
| Roof | Vegetable | 92.4 | 7.3 | 0.2 |
|  | Sheet metal | 17.2 | 74.6 | 8.2 |
|  | Tile | 26.3 | 65.8 | 7.9 |
|  | Outdoor-Kitchen | 49.8 | 48.0 | 2.2 |
| Kitchen-floor | Soil | 71.7 | 27.5 | 0.7 |
|  | Plank | 36.1 | 61.9 | 2.1 |
|  | Tiles | 0.0 | 34.3 | 65.7 |
|  | Cement | 47.0 | 46.3 | 6.7 |
| Bedroom-floor | Soil | 93.3 | 6.7 | 0.0 |
|  | Plank | 48.2 | 47.4 | 4.5 |
|  | Tiles | 6.2 | 9.4 | 84.4 |
|  | Cement | 22.9 | 71.2 | 5.9 |
| Lightening | Electricity | 1.9 | 83.0 | 15.1 |
|  | Petrol | 78.7 | 21.2 | 0.1 |
|  | Candle | 27.0 | 70.4 | 2.5 |
|  | Battery-powered | 73.2 | 26.8 | 0.0 |
| Combustion | Electricity | 0.0 | 50.0 | 50.0 |
|  | Gas | 0.0 | 7.7 | 92.3 |
|  | Coal | 21.2 | 70.3 | 8.5 |
|  | Firewood | 100.0 | 0.0 | 0.0 |
| Toilet | No toilet | 84.4 | 15.6 | 0.0 |
|  | Commune external | 38.2 | 60.6 | 1.2 |
|  | Individual external | 30.8 | 64.2 | 5.0 |
|  | Toilet inside | 0.0 | 39.6 | 60.4 |
| Main assets | Computer | 0.0 | 23.5 | 76.5 |
|  | Cars | 6.2 | 35.8 | 58.0 |
|  | Internet access | 0.0 | 7.3 | 92.7 |
|  | Refrigerator | 0.0 | 56.1 | 43.9 |
